# *HABP2* p.G534E variant in patients with family history of thyroid and breast cancer

**DOI:** 10.18632/oncotarget.16639

**Published:** 2017-03-29

**Authors:** Maisa Pinheiro, Sandra Aparecida Drigo, Renata Tonhosolo, Sonia C.S. Andrade, Fabio Albuquerque Marchi, Igor Jurisica, Luiz Paulo Kowalski, Maria Isabel Achatz, Silvia Regina Rogatto

**Affiliations:** ^1^ CIPE - International Research Center, A. C. Camargo Cancer Center, Sao Paulo, SP, Brazil; ^2^ Department of Urology, Faculty of Medicine, São Paulo State University, UNESP, Botucatu, SP, Brazil; ^3^ Department of Genetics and Evolutionary Biology, University of Sao Paulo, USP, Sao Paulo, SP, Brazil; ^4^ Princess Margaret Cancer Centre, University Health Network and The University of Toronto, Toronto, ON, Canada; ^5^ Institute of Neuroimmunology, Slovak Academy of Sciences, Bratislava, Slovakia; ^6^ Department of Head and Neck Surgery and Otorhinolaryngology, A. C. Camargo Cancer Center, Sao Paulo, SP, Brazil; ^7^ Division of Cancer Epidemiology and Genetics, National Cancer Institute/National Institutes of Health, Bethesda, MD, USA; ^8^ Department of Clinical Genetics, Vejle Hospital, Institute of Regional Health Research, University of Southern Denmark, Vejle, Denmark

**Keywords:** breast cancer, thyroid cancer, genetics, molecular markers, hereditary tumors

## Abstract

Familial Papillary Thyroid Carcinoma (PTC) has been described as a hereditary predisposition cancer syndrome associated with mutations in candidate genes including *HABP2*. Two of 20 probands from families with history of PTC and breast carcinoma (BC) were evaluated by whole exome sequencing (WES) revealing *HABP2* p.G534E. Sanger sequencing was used to confirm the involvement of this variant in three families (F1: 7 relatives; F2: 3 and F3: 3). The proband and his sister (with no malignant tumor so far) from F1 were homozygous for the variant whereas one relative with PTC from F2 was negative for the variant. Although the proband of the F3 with PTC was *HABP2* wild type, three relatives presented the variant. Five of 170 healthy Brazilian individuals with no family history of BC or PTC and three of 50 sporadic PTC presented the p.G534E. These findings suggested no association of this variant with our familial PTC cases. Genes potentially associated with deregulation of the extracellular matrix organization pathway (*CTSB*, *TNXB*, *COL4A3*, *COL16A1*, *COL24A1*, *COL5A2*, *NID1*, *LOXL2*, *MMP11*, *TRIM24* and *MUSK*) and DNA repair function (*NBN* and *MSH2*) were detected by WES, suggesting that other cancer-associated genes have pathogenic effects in the risk of familial PTC development.

## INTRODUCTION

Thyroid Carcinoma (TC) is a common endocrine neoplasia corresponding to 1% of all cancers worldwide [1, 2]. Non-Medullary Thyroid Carcinoma (NMTC), which includes Papillary Thyroid Carcinoma (PTC) and Follicular Thyroid Carcinoma (FTC), is the most common tumor involving the thyroid gland, the former being responsible for 80-95% of all cases [3, 4].

Family history of NMTCs has been associated with well known Hereditary Cancer Predisposition Syndromes (HCPS) including Cowden, Familial Adenomatosis Polyposis/Gardner, Carney Complex type 1, Werner and DICER1 syndromes, which were associated with mutations in *PTEN*, *APC*, *PRKAR1A*, *WRN* and *DICER1* genes, respectively [5, 6]. Although TC has been reported in families with Lynch and Li-Fraumeni syndromes, its inclusion in the spectrum of tumors of these disorders remains to be clarified [7–11].

The predominance of PTC in families having first or second degree relatives affected by the disease has been described as a particular clinical entity suggestive of a heritable component. Based on this premise, new candidate genes have been reported as associated with familial PTC including *HABP2, SRGAP1*, *PARP4* and *SRRM2* [12–15].

Gara et al. (2015) reported a novel non-synonymous p.G534E variant in the Hyaluronan Binding Protein 2 gene (*HABP2*, which encodes for a member of the peptidase S1 family of serine proteases) associated with familial NMTC. Based on the loss of function, the authors suggested that *HABP2* is a tumor suppressor gene with dominant-negative effect [15]. Zhang and Xing reported the presence of this variant in heterozygous form in six individuals with PTC from four kindred [16]. Contrary to these reports, Tomsic et al. (2016) evaluated the variant *HABP2* p.G534E in 179 individuals with family history of NMTC, 1160 sporadic PTC and 1395 controls showing an allele frequency of 0.61, 0.8 and 0.87, respectively. In six of eight families, the authors did not find co-segregation of the mutated allele and phenotype [17].

An intensive debate has been raised in literature [18–20] based on the allele frequency described as common in several populations as well as in public and in house databases. In highly inbred Middle Eastern population, Alzahrani et al. (2016) described the absence of mutations in familial NMTC and only one of 509 sporadic cases harbored the variant [21]. In Hispanic individuals (281 NMTC and 1105 population-matched controls) Bohórquez et al. (2016) reported that the *HABP2* p.G534E was not significantly associated with cancer susceptibility [22]. In British population, no significant association was found between the p.G534E and NMTC [23]. Similarly, the variant was not associated with PTC neither in Spanish nor Australian families [24, 25]. According to Carvajal-Carmona et al. (2016), the high allele frequency is a plausible explanation for the identification of carriers in different populations [26].

In this study we identified the *HABP2* p.G534E mutation in three Brazilian families with history of PTC and BC. We also assessed the presence of the variant in sporadic PTC cases as well as in healthy Brazilian individuals. Extracellular matrix organization pathway and DNA repair function might be potentially deregulated and contribute to the risk of developing thyroid familial cancer.

## RESULTS

### Detection of *HABP2* p.G534E variant in patients with familial history of NMTC and BC

#### Family 1

**Figure 1:**
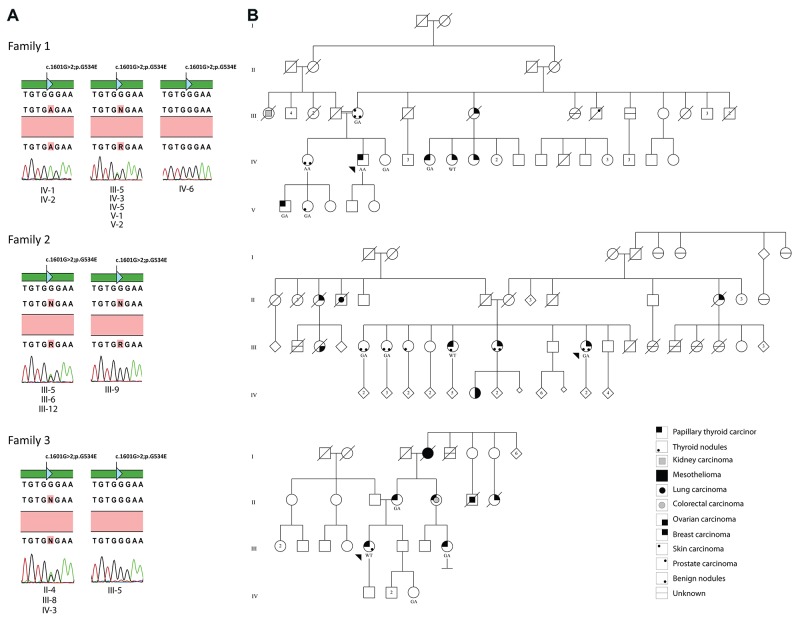
Segregation analysis and *HABP2* p.G354 status in Family 1, 2 and 3 WT. Homozygous for the wild-type allele. GA. Heterozygous. AA. Homozygous for the p.G354E variant. Circles and squares represent female and male members, respectively. The probands are indicated by black arrows. Deceased members are represented by diagonal lines. **(A)** Eletroferograms representing *HABP2* mutation status for each tested individual. **(B)** Pedigree from families 1, 2 and 3.

The male patient diagnosed with PTC (Case 1: IV-2) was detected as homozygous for the *HABP2* p.G534E variant by exome sequencing analysis and confirmed by Sanger sequencing. He had no clinical or pathological features of tumor aggressiveness (Figure 1, Supplementary Table 1). Seven family members (4 affected and 3 unaffected) were screened by Sanger sequencing revealing six positive individuals for the *HABP2* p.G534E (Figure 1, Supplementary Table 1). The sister of the patient (IV-1 with history of thyroid goiter and benign endometrial polyp) was homozygous for the mutation (Figure 1). The variant in heterozygous form was detected in his mother with skin carcinoma (III-5), the sister diagnosed with PTC (IV-5), two relatives with PTC (IV-5 and V-1) and two relatives unaffected by cancer (IV-3 and V-2) (Figure 1). His cousin diagnosed with BC (IV-6) was negative for p.G534E (Figure 1B).

#### Family 2

A woman with history of invasive ductal BC (Case 2; III-12) presented the *HABP2* p.G534E variant detected by WES. She was also diagnosed with four different benign tumors in a period of six years, including thyroid goiter, breast nodules, skin nodules and colorectal polyps. Sanger sequencing confirmed the presence of this variant in the index case and in two of three sisters (III-5: thyroid colloid goiter and colorectal hyperplasic polyp; III-6: thyroid follicular adenoma and benign breast nodule) (Figure 1A). The only sister diagnosed with PTC (III-9) was negative for p.G534E (Figure 1B, Supplementary Table 1).

#### Family 3

Sanger sequencing of *HABP2* exon 13 was carried out in the index patient (Case 3) and three relatives from family 3. The 40 year-old female proband (III-5) diagnosed with PTC was negative for p.G534E mutation. Three relatives tested were positive for the same variant (Figure 1, Supplementary Table 1) including the mother (II-4: PTC), the female cousin (III-8: PTC) and the proband's niece (IV-3: no history of cancer). The index patient had also breast cancer family history from maternal lineage (II-7) (Figure 1). Despite not being possible to assess her aunt (II-5), diagnosed with PTC and colorectal carcinoma, we may conclude she is positive for p.G534E since her daughter (III-9) carried this allele (Figure 1).

### Sporadic PTC and healthy Brazilian individuals

The DNA of blood samples from both, 170 healthy individuals and 50 patients with sporadic PTC, were screened by Sanger sequencing to investigate the *HABP2* exon 13. Five healthy individuals were heterozygous for the variant, which represents an Allele Frequency (AF) of 0.0147. In PTC samples the AF was 0.04: two tumors were heterozygous and one was homozygous for p.G534E. Clinical features from sporadic PTC are presented in Supplementary Table 2.

### Variant prioritization and protein-protein interaction network

Our results suggested no co-segregation of the *HABP2* p.G534E mutated allele and the phenotype. Furthermore, the frequency of this alteration in sporadic PTC and healthy Brazilian individuals indicated that this allele is common in Brazilian individuals. Based on these findings, the *SRGAP1*, *PARP4* and *SRRM2* candidate genes related to familial NMTC were also investigated. Although non-synonymous mutations have been found, none of them were identical to those previously reported.

Case 1 presented 14 variants (*SRGAP1*: p.L953V; *SRRM2*: p.P804T, p.R1934H; *PARP4*: p.I1564T, p.L1550P, p.S1459Y, p.S1394A, p.P1328T, p.G1280R, p.R1108C, p.L1080R, p.V1065A, p.M936T, p.A899T), three of them called deleterious in at least one bioinformatics prediction database for pathogenicity (dbNSFP, Supplementary Table 3). Two of them were classified as rare mutations according to 1000 Genomes [27] and 6500 Exomes [28]: *SRGAP1* p.L953V (MAF 0.016) and *SRRM2* p.R1934H (not described).

The index patient 2 showed 12 non-synonymous mutations (*SRRM2*: p.P804T; *PARP4*: p.I1564T, p.L1550P, p.S1459Y, p.S1394A, p.R1108C, p.L1080R, p.V1065A, p.M936T, p.S873N, p.I81V, p.A899T). Two of them, mapped in *PARP4* gene, were predicted as pathogenic in at least one dbNSFP annotation: p.A899T (MAF: 0.0323) and p.I81V (MAF: 0.6792). However, the p.I81V is considered common in populational public databases [27, 28].

**Figure 2:**
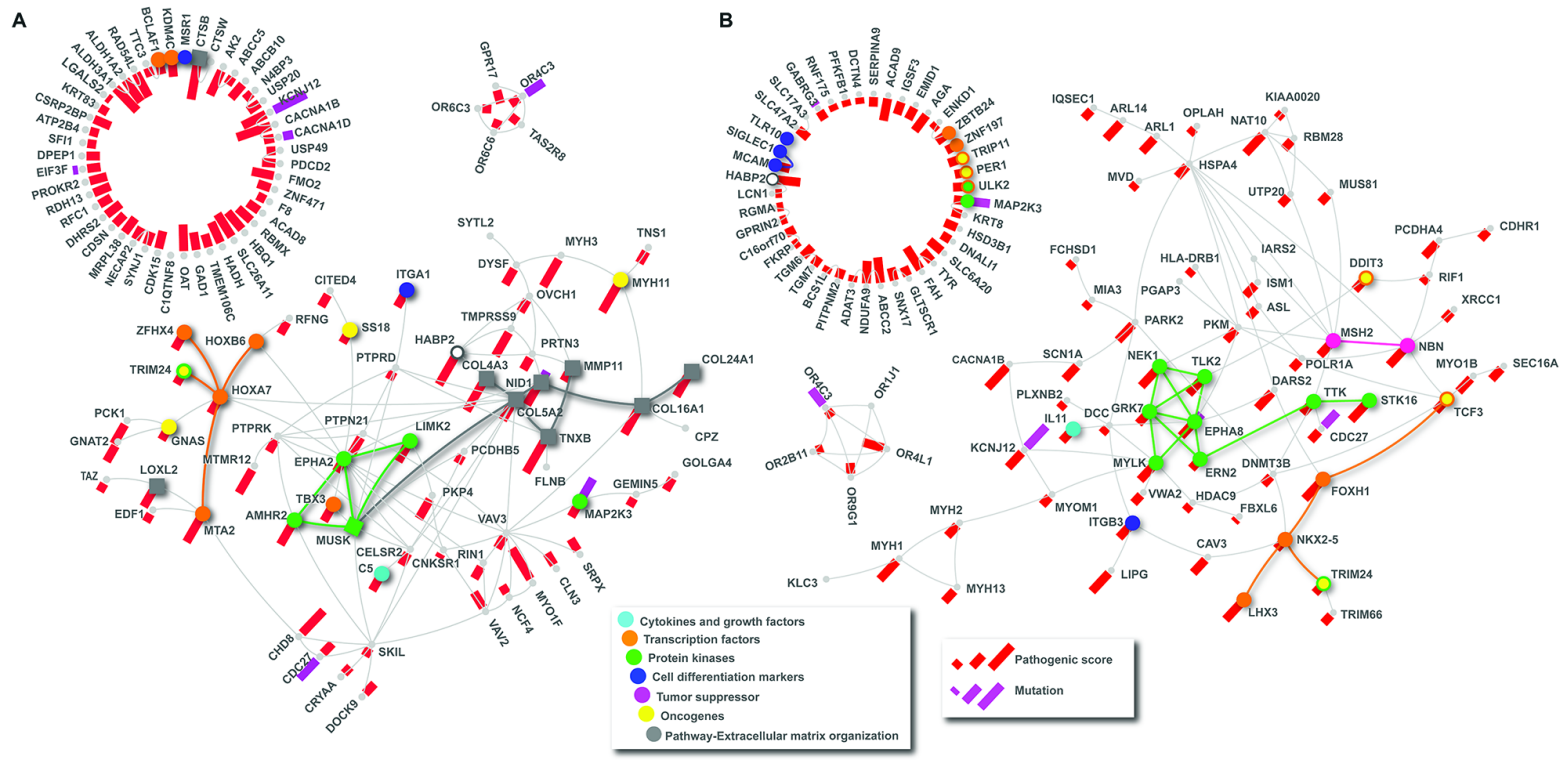
Protein-protein interaction (PPI) network analysis PPI networks based on mutated genes detected by WES analysis, which were classified as deleterious in at least three dbNSFP pathogenicity predictions, and their interactions partners identified in IID and visualized with NAViGaTOR. Highlighted diamonds represent mutated genes classified according to MSigDB. Squares represent genes enriched for Extracellular Matrix Organization pathway. Pathogenic score and mutation is highlighted by spokes as per legend. **(A)** Mutated genes identified in the proband 1. **(B)** Mutated genes identified in the index patient 2.

To gain further insight into the function of other putative candidates associated with familial NMTC, we scrutinized WES data. Rare variants reported with MAF ≤ 0.01 in 1000 Genomes phase 3 [27] and 6500 Exomes [28] were prioritized. Variants ranked per pathogenicity prediction, using dbNSFP, are described in details in Supplementary Table 4. A comprehensive pathway enrichment analysis using genes that harbor mutations predicted as deleterious in at least three dbNSFP was performed using KOBAS tool [29] and pathDIP [30]. Extracellular matrix organization pathway was significantly altered for case 1 (P=5.3×10^-5^; FDR=0.016). From higher to lower pathogenic degree, the mutated genes in this pathway included *CTSB*, *TNXB*, *COL4A3*, *COL16A1*, *COL24A1*, *COL5A2*, *NID1*, *LOXL2*, *MMP11*, *TRIM24* and *MUSK*, (Figure 2A). Although not enriched in the same pathway, *HABP2* was related to extracellular matrix degradation interacting with *COL4A3*. In addition, cancer related genes [31] as well as cytokines and growth factors, protein kinases, transcription factors and oncogenes presented pathogenic mutations. From these, *MTA2*, *MYH11* and *AMHR2* have highly pathogenic mutations displayed by pathogenic score (Figure 2A).

Similar analysis was performed for the altered genes found in the index Case 2. Although no significantly enriched pathway was deregulated, two tumor suppressor genes, *MSH2* and *NBN* were mutated (Figure 2B). Although predicted as pathogenic, the p.G322D *MSH2* variant (c.965G>A) is classified as benign for the Lynch Syndrome phenotype (ClinVar Variation ID: 1762). Currently, the alteration c.596C>T; p.P199L in *NBN* gene has not been described in public databases. A considerable number of genes harboring pathogenic variants that codify protein kinases was detected including *ERN2*, *MAP2K3*, *EPHA8*, *STK16*, *GRK7*, *MYLK*, *TTK*, *NEK1*, *ULK2*, *TRIM24* and *TLK2*. Alterations in cell differentiation markers, cytokines and growth factors, transcription factors and oncogenes were also detected (Figure 2B). The protein-protein interaction (PPI) network analysis using IID [32] revealed connections among *FOXH1*, *LHX3*, *NBN*, *NEK1*, *STK16*, which could be relevant in the phenotype (Figure 2B).

A comprehensive pathway enrichment analysis was performed using all proteins from Figure 2. The combination of the two cases identified their physical protein interactions using IID [32] (Figure 2; Supplementary Table 5). The resulting proteins were used to query pathDIP [30] that identified 31 significantly enriched pathways (p<0.043, FDR: BH-method; Supplementary Table 5), including leptin (p<0.0059), integrins (p<0.041), ECM-receptor interaction (p<0.036), EGFR and ERBB4 (p<0.043). Importantly, the most enriched terms in these pathways included focal-adhesion, EGF, Estrogen, PI3K, and prolactin (Supplementary Table 5).

## DISCUSSION

Two probands with personal and or family history of BC and PTC evaluated by exome sequencing analysis presented *HABP2* p.G534E variant. The probands and several relatives from these two families diagnosed with PTC, thyroid goiter, thyroid nodules, BC and other tumors were tested using Sanger sequencing method. Our data revealed the absence of complete co-segregation between the mutation and the phenotype. Two individuals from Family 1, including one diagnosed with PTC and his healthy sister, harbored homozygous p.G534E mutation as result of consanguineous marriage. The homozygous cases have no clinical or pathological differences compared with heterozygous cases. The third family evaluated only by Sanger sequencing revealed three of four individuals with the p.G534E variant; however, the index patient who had PTC was negative for the mutation. Furthermore, two healthy individuals from families 1 and 3 (both, IV-3) were positive for the mutation. Overall, these findings give additional support to the absence of the association between NMTC and *HABP2* p.G534E variant.

Interestingly, we have identified patients diagnosed with BC positive for the *HABP2* p.G534E mutation, which is additional evidence that this variant is not associated with thyroid disease. To our knowledge, breast cancer has not been described as associated with p.G534E mutation.

To investigate the *HABP2* p.G534E allele frequency in Brazilian population, 170 healthy individuals were evaluated and five of them were heterozygous for the mutation (AF = 0.0147). The ethnicity of Brazilian individuals is questionable mostly by the high level of miscegenation; however, the Brazilian genetic background also derives from the European population [33, 34]. According to the last update of 1000 Genomes project Phase 3 [27] and considering European allele frequency, the minor allele frequency of p.G534E is 0.027, which is not significantly different from the Brazilian individuals tested in our study (Fisher's exact test, P = 0.3). Similarly, no significant results were found in the comparison with the European allele frequency in ExAC database (unfinished) [35], in which p.G534E allele frequency is 0.033 (Fisher's exact test, P = 0.065).

The *HABP2* mutation allele frequency was 0.04 in 50 sporadic PTC samples, which is consistent with data reported by TCGA (0.047). The comparison between healthy Brazilian individuals and sporadic PTC cases revealed no significant difference (Fisher's exact test, P = 0.124). Based on these findings, Brazilian allele frequency is concordant with public databases and no association of p.G534E with sporadic PTC was found.

Since Gara et al. (2015) reported the *HABP2* p.G534E as a candidate to hereditary NMTC [15], several studies have been reported aiming to establish the involvement of this mutation as a non random event in different populations. Except for Zhang et al. (2016) [16], these studies have failed to prove the association between NMTC and *HABP2* variant, including our study. Data reported in Spanish population [22, 24], highly inbred Saudi Arabian population [21], United Kingdom and Ireland [23], as well as patients followed by Ohio State University, United States [17], have demonstrated no involvement of p.G534E as a predisposition gene in thyroid tumors.

In the past few years, new variants associated with familial NMTC have been reported including *SRGAP1* (Q149H and R617C), *SRRM2* (p.S346F) and *PARP4* (p.G496V and p.T1170I) [12–14]. These specific variants were not detected in our probands (cases 1 and 2). However, the rare mutation *SRRM2* p.R1934H (pathogenic in four pathogenicity predictions and no described in dbSNP so far) was detected in the index Case 1 (Supplementary Table 3). Interestingly, the allele frequency of this variant was 2.594×10^-4^ and 2.999×10^-5^ in Latino and European populations (ExAC project, not finished) [35], respectively. Further studies are necessary to better evaluate the involvement of the *SRRM2* in familial NMTC.

A considerable number of alterations with potential to modify the effect of tumor suppressor genes have been described [36]. In addition, the combination of different mutations in cancer predisposition genes has implicated in phenotype overlapping and a synergic interaction, where several loci may contribute with the phenotype [37]. For instance, a combination of papillary thyroid carcinoma and paraganglioma in the same patient revealed alterations in *PTEN* and *SDHC* genes, leading to Cowden syndrome and pheochromocytoma–paraganglioma diagnosis [38]. A recent study suggested that the mutation background of an individual might modulate the severity of the Mendelian diseases [39]. In our study, no classical mutations associated with hereditary cancer syndromes were identified, however several rare and predicted as pathogenic mutations were detected in the Cases 1 and 2. For instance, Case 1 harbors pathogenic mutations in genes involved in extracellular matrix organization pathway, similar with *HABP2* protein function [40]. The extracellular matrix proteins are involved in angiogenesis, cell growth, fibrosis, cell signaling and disrupting of tissue morphogenesis [41]. In thyroid cancer, dysfunction of *COLL1* and *LOX* genes, promoted by *BRAF* and *PTEN* knockdown, were associated with ripping stiffen of Col1 matrix, enhancing cell motility and tumor progression [42]. In our analysis, *CTSB* gene harbors a mutation predicted as highly pathogenic (Figure 2A). Protein encoded by *CTSB* regulates negatively Wnt/β-catenin signaling pathway [43] related with tumor development [44]. Extracellular matrix is essential for breast normal function (as mammary gland remodeling before and after lactation) and tumor development, progression and metastasis, as its composition differs between metastatic and non-metastatic breast tumor stages [45]. Inhibitors of extracellular components as well as integrins, proteoglycans, metalloproteinase, collagens and C1-peptidase protein families have demonstrated promising pre-clinical results in breast cancer [45]. For instance, cathepsin B (*CTSB* gene, reported as mutated in our study) inhibition has been reported as associated with reduced bone metastasis in breast cancer from mice [46].

Although predicted pathogenic alterations were found involving *MSH2* and *NBN*, no significantly enriched pathway was found for index Case 2. *MSH2* p.G322D variant is described as benign in the Lynch Syndrome diagnosis (ClinVar Variation ID: 1762) [47]. Recently, the *MSH2* p.G322D variant was associated with breast cancer risk in Polish women [48]. *NBN* is DNA repair family gene, involved in the *MRE11/RAD50/NBN* complex, with nine variants (rs1805794, rs1805790, rs36226237, rs924, rs376639, rs1061302, rs1063054, rs2735383, rs805794) described as associated with high cancer risk [49]. Recently, Kraus et al. investigate the mutational status of 581 BC patients using a panel-based screening of 14 breast and ovary susceptibility genes, including *NBN*. One patient presented a deleterious variant c.657_661del5 of *NBN* [50]. In addition, the c.657del5 variant in the NBN gene was reported as associated with pancreatic cancer predisposition [51]. Differently of these reports, we found the variant c.596C>T p.P199L in our case 2. Eleven mutated genes classified as protein kinases were also identified in our Case 2 (Figure 2B). Interestingly, a group of proteins highly connected and with the same function might lead to cellular dysfunction and pre-cancer stages. In our study, the index patient 2 was diagnosed with BC, benign breast nodule, thyroid nodules and colorectal polyps. The presence of mutations in *MSH2* and *NBN* has the potential to increase the risk of the patient developing multiple primary tumors.

A comprehensive pathway enrichment analysis using pathDIP and proteins from the network (Figure 2) revealed 31 significantly enriched pathways (p<0.043), including leptin, integrins, interleukins, ECM-receptor interaction, EGFR and ERBB4. Interestingly, the most enriched terms in the titles of these pathways included focal-adhesion, EGF, Estrogen, PI3K, and prolactin (Supplementary Table 5). In the current study, we focused on comprehensive pathway enrichment analysis. In the future, using additional patient cases and larger PPI network, we could use additional tools that combine mutation data (such as HotNet [52]) or arbitrary omics data (e.g., KeyPathwayMiner [53], NBS [54], MUFFIN [55], VarWalker [56]) with signaling networks.

In conclusion, no co-segregation of the *HABP2* p.G534E was found in three families with NMTC and breast cancer. The variant is not significantly associated with PTC in Brazilian population and showed similar allele frequency in tumor samples and healthy individuals. Our data also refute the dominant negative effect of this variant as proposed by Gara et al. (2015) [15]. Alterations reported here may contribute to high risk of NMTC. A limitation of our study was the impossibility to evaluate the exome of all affected family members, compromising the identification of other putative *locus* of predisposition that segregate with the phenotype. Our results point out that the *HABP2* p.G534E mutation is not associated with hereditary NMTC. Genetic mechanisms involved in familial NMTC remain elusive and further investigation is necessary.

## MATERIALS AND METHODS

### Ethics statement

The use of the clinical samples was approved by the Institutional Human Research Ethics Committees at A.C. Camargo Cancer Center, Sao Paulo and Faculty of Medicine, UNESP, Botucatu, Sao Paulo, Brazil (FMB-PC-197/2012; CEP1175/08ext). Informed consent was obtained from all patients, and the experiments were performed in accordance with relevant guidelines and regulations.

### Patients and clinical samples

Twenty index cases with personal and family history of NMTC, thyroid disease as well as breast carcinoma were evaluated by whole exome sequencing. We focused this study on two patients that presented *HABP2* p.G534E variant, one diagnosed with invasive ductal breast carcinoma and the other with PTC. Family members from index patient 1 (7 relatives) and 2 (three relatives) were included in this study. Although these patients presented tumors frequently described in the Cowden Syndrome (CS) spectrum, none of them fulfilled the clinical criteria for CS. In addition, the index patients were negative for pathogenic mutations in *PTEN*, *BRAC1*, *BRCA2* and *TP53* genes. A third index patient diagnosed with PTC, with family cancer history of PTC and BC and three relatives, were also investigated for *HABP2* status using Sanger sequencing. All patients were prospectively followed by the Department of Oncogenetics, A.C. Camargo Cancer Center, Sao Paulo, Brazil. Clinical features and family history of all individuals are presented on Supplementary Table 1.

Index Case 1was diagnosed with PTC at 41-years-old. His familial history included a nephew diagnosed with PTC (27 years old), an aunt diagnosed with BC (90 years old) and skin carcinoma (87 years old) who had three daughters, one diagnosed with PTC (55 years old), and two with BC (at age 60 and 61, respectively) (Figure 1B, Supplementary Table 1). The proband's mother developed a skin tumor (75 years old) and thyroid colloid goiter (67 years old). She was married with a first-degree cousin.

Index Case 2 presented a BC (46 years old) and a history of several benign lesions (benign nodules from breast, skin, thyroid and colorectal polyp). Her sisters presented history of cancer: one with papillary thyroid carcinoma (66 years old) and the other with BC (58 years old). Her niece was diagnosed with breast and ovary carcinomas (47 years-old); two aunts with BC, beyond other malignancies in the family (Figure 1B).

The proband from the third family was diagnosed with PTC (40 years old) concurrent with breast benign nodules. Her family history encompasses a mother with PTC (40 years old), a female cousin with PTC (38 years old), an aunt with colorectal cancer (40 years old) and PTC (50 years old), and a second-degree female cousin diagnosed with BC (50 years old). This family also presented individuals with throat, pleura and other tumors types in the maternal lineage (Figure 1B, Supplementary Table 1).

A cohort of 170 healthy individuals (84 males and 86 females) was recruited at the same hospital and screened for the variant p.G534E. These individuals were cancer-free so far, with no known thyroid diseases or family history of cancer. The median age of this set of cases was 49 years (range 21-87 years old). Fifty sporadic PTC (11 male and 39 female) with median age of 40 years (range 21-75 years old) were also assessed. These patients have no family history of cancer. Clinical and histological characteristics of the sporadic PTC are detailed in Supplementary Table 2.

### Genomic DNA extraction

Genomic DNA was extracted from blood leukocytes using Qiacube DNA Blood kit (Qiagen, Valencia, CA) for two index patients and all healthy individuals. Family members from *HABP2* p.G534E positive patients, as well as the individuals for family 3, had their DNA extracted from saliva using Oragene-DNA (DNA Genotek, Ottawa, CA). Gentra Puregene Tissue Kit (Qiagen, Valencia, CA) was used to obtain DNA from fresh frozen PTC tissue samples. The DNA extraction followed the manufacturer's recommendations.

### Whole exome sequencing (WES) analysis

Whole exome libraries were prepared with Exome Nextera Enrichment kit-Illumina (San Diego, California, USA) with 62 Mb target regions. Paired-end sequencing of 100 bp was carried out in HiSeq2500 Illumina (San Diego, California, USA), at the Center for Functional Genomics core facility, ESALQ-University of Sao Paulo, Piracicaba, Brazil.

Raw reads data were trimmed with Seqyclean software [57]. Paired-end reads were aligned to UCSC hg19 using Botiwe2 [58], in local-alignment mode. WES analysis achieved coverage of 53x. Duplicate reads were marked using the MarkDuplicates utility from Picard [59] to filter out PCR artifacts. Variants were detected with GATK [60] and SAMtools [61]. ANNOVAR [62] was used for data annotation.

Exonic alterations were prioritized according to their function focusing on frameshift, splice-site and non-synonymous variants. Data were compared with 1000 Genomes phase 3 [27] and 6500 Exomes [28] and variants reported with an allele frequency ≤0.01 were selected. After the annotation with dbNSFP database [63], the alterations classified as deleterious in at least three pathogenicity predictions were selected to perform pathway enrichment and network analysis. Pathways enrichment analysis was performed using KOBAS 2.0 tool [29]. Selected variants were then used to identify biological context by querying protein-protein interaction (PPI) database Interologous Interaction Database v. 2016-03 [32] (http://ophid.utoronto.ca/iid). The resulting network was visualized in NAViGaTOR v3 [64] (http://ophid.utoronto.ca/navigator).

To elucidate biological functions of the protein network (Figure 2), we performed a comprehensive pathway enrichment analysis. The Pathway Data Integration Portal (pathDIP) was used to perform enrichment analysis across pathways from 20 major pathway databases (pathDIP ver. 2.5; [30]). We considered literature curated gene: pathway memberships and those predicted according to experimentally detected protein-protein interactions (including interactions experimentally detected between orthologues plus FpClass [65] interactions with minimum confidence level for predicted associations equal 0.95 (for more details see pathDIP documentation in http://ophid.utoronto.ca/pathDIP).

### Data confirmation and validation

The presence of the variant p.G534E detected by WES was confirmed by Sanger re-sequencing in the two index cases, their family members and in the family 3 members. For the *HABP2* exon 13 amplification the following primers were constructed: forward primer 3’-CCCTGACACCCCCTGGAGAG-5’; and reverse primer 3’-GCTCTGGAGGTGTCCATTGT-5’ (Gara et al., 2015). Polymerase chain reaction was performed according to standard protocols using Platinum Taq DNA Polymerase (Life Technologies, Carlsbad, California, USA) with the following conditions: 2 min at 94°C; followed by 35 cycles of 45 sec at 94°C, 30 sec at 63°C and 45sec at 72°C; followed by 10 min at 72°C for final extension. Sanger sequencing was carried out using BigDye terminator v3.1 and ABI Prism 3130XL sequencer, both from Applied Biosystem (Foster City, California, USA).

## SUPPLEMENTARY MATERIALS TABLES











## Abbreviations

PTC: Papillary Thyroid Carcinoma
WES: Whole Exome Sequencing
TC: Thyroid Cancer
NMTC: Non-Medullary Thyroid Carcinoma
FTC: Follicular Thyroid Carcinoma
HCPS: Hereditary Cancer Predisposition Syndromes
BC: Breast Carcinoma
KOBAS: KEGG Orthology-Based Annotation System
AF: Allele Frequency
MAF: Minor Allele Frequency
dbNSFP: Single Nucleotide Polymorphism Database
PPI: Protein-Protein Interaction
ExAC: Exome Aggregation Consortium
TCGA: The Cancer Genome Atlas
CS: Cowden Syndrome
