# Supplementary material for: HABP2 p.G534E variant in patients with family history of thyroid and breast cancer
Source: Oncotarget. 2017 Mar 29;8(25):40896–905. doi: 10.18632/oncotarget.16639 (PMC5522276; doi:10.18632/oncotarget.16639)
Supplement: Supplementary file 1 [file oncotarget-08-40896-s001.pdf]

HABP2 p.G534E variant in patients with family history of thyroid and breast cancer

SUPPLEMENTARY TABLES

Supplementary Table 1: Clinical features and *HABP2* p.G534E status for patients from families 1, 2 and 3

See Supplementary File 1

Supplementary Table 2: Clinical characteristics of 50 sporadic PTC patients tested for HABP2 status

Characteristics	Nº of Patients (%)
**Age (years)**	median 40 (21-75)
**Gender**	
Female	39 (78)
Male	11 (22)
**Tumor dimension (cm) of**	median 1.5 (0.3-5.5)
**predominant variant**	
Classic	32 (64)
Follicular	16 (32)
Rare	2 (4)
**Perineural invasion**	
No	45 (90)
Yes	4 (8)
Ni	1 (2)
**Extrathyroidal extension**	
No	31 (62)
Yes	19 (38)
Ni	1 (2)
**Lymph node metastasis**	
No (cN0, pN0)	34 (68)
Yes (pN1)	16 (32)

Ni: not informed.

Supplementary Table 3: Variants identified in genes recently reported as associated with NMTC

See Supplementary File 2

Supplementary Table 4: Variants ranked per pathogenicity prediction using dbNSFP

See Supplementary File 3

Supplementary Table 5: Pathway enrichment analysis and physical proteins interactions

See Supplementary File 4
