# Supplementary material for: HABP2 p.G534E variant in patients with family history of thyroid and breast cancer
Source: Oncotarget. 2017 Mar 29;8(25):40896–905. doi: 10.18632/oncotarget.16639 (PMC5522276; doi:10.18632/oncotarget.16639)
Supplement: Supplementary file 2 [file oncotarget-08-40896-s002.docx]

**SUPPLEMENTARY TABLES**

**Supplementary Table 1.** Clinical features and *HABP2* p.G534E status for patients from families 1, 2 and 3.

| **Patient** | **Gender** | | | **Age** | **Diagnosis** | **Surgical intervention** | **Treatment** | **TNM** | **Tumor multifocality** | **Extrathyroidal extension** | **Vascular invasion** | **Peri invasion** | **G534E status** |
| --- | --- | --- | --- | --- | --- | --- | --- | --- | --- | --- | --- | --- | --- |
| **Family 1** | | |  |  |  |  |  |  |  |  |  |  |  |
| IV-2 | M | | | 41 | Papillary thyroid | Thyroidectomy | Radioiotherapy | T1N0M0 | YES | NO | NO | NO | HOM |
|  |  | | | 41 | Thyroid colloid goitre |  |  |  |  |  |  |  |  |
| IV-1 | F | | | 62 | Thyroid goitre |  |  |  |  |  |  |  | HOM |
|  |  | | | 54 | Uterus benign nodule |  |  |  |  |  |  |  |  |
| IV-3 | F | | | 58 | No cancer |  |  |  |  |  |  |  | HET |
| IV-5 | F | | | 55 | Papillary thyroid |  |  |  |  |  |  |  | HET |
| IV-6 | F | | | 60 | Breast carcinoma |  |  |  |  |  |  |  | WT |
| III-5 | F | | | 67 | Thyroid colloid goitre |  |  |  |  |  |  |  | HET |
|  |  | | | 75 | Skin basal cell carcinoma |  |  |  |  |  |  |  |  |
|  |  | | | 79 | Colon benign nodule |  |  |  |  |  |  |  |  |
|  |  | | | 81 | Stomach benign nodule |  |  |  |  |  |  |  |  |
| V-1 | M | | | 27 | Papillary thyroid | Thyroidectomy |  |  | YES | NO | NO | NO | HET |
| V-2 | F | | | 24 | Thyroid colloid goitre |  |  |  |  |  |  |  | HET |
| **Family 2** | |  | |  |  |  |  |  |  |  |  |  |  |
| III-12 | F | | | 46 | Breast carcinoma (left) | Mastectomy | Radiotherapy, chemotherapy, hormone therapy |  |  |  |  |  | HET |
|  |  | | | 46 | Breast benign nodule (right) |  |  |  |  |  |  |  |  |
|  |  | | | 48 | Skin benign nodule |  |  |  |  |  |  |  |  |
|  |  | | | 51 | Colorectal polyps | Polypectomy |  |  |  |  |  |  |  |
|  |  | | | 52 | Thyroid nodules |  |  |  |  |  |  |  |  |
| III-9 | F | | | 63 | Thyroid colloid goitre |  |  |  |  |  |  |  | WT |
|  |  | | | 66 | Papillary thyroid | Thyroidectomy | Radioiodotherapy | T3N0M0 | NO | NO | NO |  |  |
| III-6 | F | | | 54 | Thyroid follicular adenoma | Prophylactic thyroidectomy |  | - |  |  |  |  | HET |
|  |  | | | 63 | Thyroid colloid goitre |  |  |  |  |  |  |  |  |
|  |  | | | 73 | Breast fibradenoma |  |  |  |  |  |  |  |  |
|  |  | | | 74 | Colorectal polyp |  |  |  |  |  |  |  |  |
| III-5 | F | | | 58 | Thyroid colloid goiter | - |  | - |  |  |  |  | HET |
|  |  | | | 61 | Thyroid nodule |  |  |  |  |  |  |  |  |
|  |  | | | 69 | Colorectal polyp |  |  |  |  |  |  |  |  |
| **Family 3** |  | | |  |  |  |  |  |  |  |  |  |  |
| III-5 | F | | | 40 | Papillary thyroid | Thyroidectomy |  |  | NO | NO | NO |  | WT |
|  |  | | | 40 | Breast nodules |  |  |  |  |  |  |  |  |
| III-8 | F | | | 38 | Papillary thyroid | Thyroidectomy |  |  |  |  |  |  | HET |
| II-4 | F | | | 40 | Papillary thyroid | Thyroidectomy |  |  |  |  |  |  | HET |
| IV-3 | F | | | 62 | No câncer | - | - | - |  |  |  |  | HET |
