# Supplementary material for: HABP2 p.G534E variant in patients with family history of thyroid and breast cancer
Source: Oncotarget. 2017 Mar 29;8(25):40896–905. doi: 10.18632/oncotarget.16639 (PMC5522276; doi:10.18632/oncotarget.16639)
Supplement: Supplementary file 3 [file oncotarget-08-40896-s003.docx]

**Supplementary Table 3:** Variants identified in genes recently reported as associated with NMTC.

| **Position** | **Reference** | **Alteration** | **Gene** | **AA Change** | **Clinvar** | **6500 ESP** | **1000 G** | **dbSNP** | **Deleterious Score dbSNFP** |
| --- | --- | --- | --- | --- | --- | --- | --- | --- | --- |
| **Index patient 1** |  |  |  |  |  |  |  |  |  |
| 64521957 | C | G | *SRGAP1* | p.L953V | NA | 0.0137 | 0.0155751 | rs113302231 | 1 |
| 2812939 | C | A | *SRRM2* | p.P804T | NA | 0.4485 | 0.414337 | rs2240140 | 0 |
| 2816330 | G | A | *SRRM2* | p.R1934H | NA | NA | NA | NA | 4 |
| 25008588 | A | G | *PARP4* | p.I1564T | NA | 0.9332 | 0.948083 | rs1372085 | 0 |
| 25008630 | A | G | *PARP4* | p.L1550P | NA | 0.9313 | 0.942692 | rs1822135 | 0 |
| 25008903 | G | T | *PARP4* | p.S1459Y | NA | NA | 0.943091 | rs9318536 | 0 |
| 25009099 | A | C | *PARP4* | p.S1394A | NA | 0.9335 | 0.948083 | rs9511259 | 0 |
| 25009297 | G | T | *PARP4* | p.P1328T | NA | 0.4059 | 0.384984 | rs1050112 | 0 |
| 25009441 | C | G | *PARP4* | p.G1280R | NA | 0.4060 | 0.385184 | rs13428 | 0 |
| 25020863 | G | A | *PARP4* | p.R1108C | NA | 0.8644 | 0.876597 | rs9318554 | 0 |
| 25021200 | A | C | *PARP4* | p.L1080R | NA | 0.9318 | 0.948283 | rs9318558 | 0 |
| 25021245 | A | G | *PARP4* | p.V1065A | NA | NA | NA | rs7334587 | 0 |
| 25027744 | A | G | *PARP4* | p.M936T | NA | 0.9333 | 0.936502 | rs4770684 | 0 |
| 25029218 | C | T | *PARP4* | p.A899T | NA | 0.6792 | 0.654153 | rs2275660 | 3 |
| **Index patient 2** |  |  |  |  |  |  |  |  |  |
| 2812939 | C | A | *SRRM2* | p.P804T | NA | 0.4485 | 0.414337 | rs2240140 | 0 |
| 25008588 | A | G | *PARP4* | p.I1564T | NA | 0.9332 | 0.948083 | rs1372085 | 0 |
| 25008630 | A | G | *PARP4* | p.L1550P | NA | 0.9313 | 0.942692 | rs1822135 | 0 |
| 25008903 | G | T | *PARP4* | p.S1459Y | NA | NA | 0.943091 | rs9318536 | 0 |
| 25009099 | A | C | *PARP4* | p.S1394A | NA | 0.9335 | 0.948083 | rs9511259 | 0 |
| 25020863 | G | A | *PARP4* | p.R1108C | NA | 0.8644 | 0.876597 | rs9318554 | 0 |
| 25021200 | A | C | *PARP4* | p.L1080R | NA | 0.9318 | 0.948283 | rs9318558 | 0 |
| 25021245 | A | G | *PARP4* | p.V1065A | NA | NA | NA | rs7334587 | 0 |
| 25027744 | A | G | *PARP4* | p.M936T | NA | 0.9333 | 0.936502 | rs4770684 | 0 |
| 25029295 | C | T | *PARP4* | p.S873N | NA | 0.1947 | 0.224641 | rs7140044 | 0 |
| 25075864 | T | C | *PARP4* | p.I81V | NA | 0.0323 | 0.0123802 | rs35200240 | 1 |
| 25029218 | C | T | *PARP4* | p.A899T | NA | 0.6792 | 0.654153 | rs2275660 | 3 |
